# Exploring Angular Distance in Protein-Protein Docking Algorithms

**DOI:** 10.1371/journal.pone.0056645

**Published:** 2013-02-21

**Authors:** Thom Vreven, Howook Hwang, Zhiping Weng

**Affiliations:** Program in Bioinformatics and Integrative Biology, University of Massachusetts Medical School, Worcester, Massachusetts, United States of America; University of Michigan, United States of America

## Abstract

We present a two-stage hybrid-resolution approach for rigid-body protein-protein docking. The first stage is carried out at low-resolution (15°) angular sampling. In the second stage, we sample promising regions from the first stage at a higher resolution of 6°. The hybrid-resolution approach produces the same results as a 6° uniform sampling docking run, but uses only 17% of the computational time. We also show that the angular distance can be used successfully in clustering and pruning algorithms, as well as the characterization of energy funnels. Traditionally the root-mean-square-distance is used in these algorithms, but the evaluation is computationally expensive as it depends on both the rotational and translational parameters of the docking solutions. In contrast, the angular distances only depend on the rotational parameters, which are generally fixed for all docking runs. Hence the angular distances can be pre-computed, and do not add computational time to the post-processing of rigid-body docking results.

## Introduction

Protein-protein interactions are important for many fundamental cellular processes, and high-throughput proteomics studies have shown that most proteins interact with other proteins. The experimental elucidation of the of protein-protein complexes structures, however, is laborious and not always successful. Starting from the unbound protein structures, computational protein-protein docking attempts to determine the structures of the bound complexes [1], [2]. This challenging problem is usually approached in a stepwise fashion. The first stage consists of a rigid-body docking run, searching the 6-dimensional (6D) rotational and translational space for binding orientations. The exhaustive search of this 6D space is time consuming, and is usually carried out with rapidly computable scoring functions and fast algorithms such as Fast Fourier transform (FFT)[3]–[6] or geometric hashing [7]. The first stage docking results may be further analyzed in a variety of ways, such as re-ranking using more sophisticated scoring functions [8]–[10], filtering [11], or clustering [12]–[14]. The second stage accounts for conformational changes of the constituent proteins upon complex formation. Such conformational changes can involve only surface side chains, the backbones of surface loops, or even entire domains [15]–[19].

We developed the ZDOCK series of programs for initial stage docking [20]–[26]. ZDOCK performs an exhaustive rigid body search in the 6D rotational and translational space. By default, three Euler angles are sampled with 6° or 15° spacing, and the three translational degrees of freedom are sampled with 1.2 Å spacing [27], [28]. For each set of rotational angles we retain only the translation with the best score, which results in thousands to tens of thousands predictions, depending on the angle spacing used. The final predictions are ranked according the ZDOCK score.

In order to cluster, prune, or post-process the large number of predictions from the rigid-body docking run, we generally need to measure the similarity between the predictions. The most common measure is the *root-mean-square distance* (RMSD), which indicates the distance between the corresponding Cα atoms (sum over *k* = 1 to *N*) of two predicted ligand orientations (*i* and *j*), keeping the receptor fixed in space:
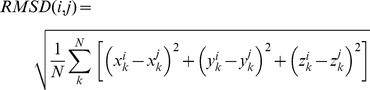
(1)


The drawback of using the RMSD as a similarity measure is that it is computationally expensive as each pair of predictions needs to be evaluated according to equation 1, which needs to be done for each docking run. In this work we explore the angular distance between predictions as an alternative for the RMSD. We define the angular distance as the angle between the rotations corresponding to two docking predictions, ignoring the translational degrees of freedom. The main advantage is that the angular distance only depends on the Euler angles of the two predictions. As rigid body docking algorithms typically sample a fixed set of angles that do not depend on the monomers or docking solutions, the angular distances can be pre-calculated and do not add computational time. This is in contrast to the RMSD’s, which need to be evaluated for each docking run. Using angular distance instead of RMSD may seem a crude approximation, as two predictions with a small angular distance may have a large RMSD and thus be very different. However, we reason that the correlation between angular distance and RMSD is largest in the local minima of well-defined energy funnels, which are the predictions that we are most interested in.

In this work we developed a two-step hybrid-resolution procedure for rigid-body docking, in which the angular distance is used to select the orientations to be explored in the second step that are in close proximity to the orientations predicted by the first step. In addition, we show that the angular distance can be used for pruning or clustering docking predictions, as well as the analysis of energy funnels.

## Methods

### Rigid-body Docking

For the rigid-body docking we used ZDOCK3, which was developed in our lab and includes the IFACE statistical pair potential [22]. The most recent implementation ZDOCK3.0.2 [23] uses a recently developed 3D convolution library for the FFT and requires an average running time per complex of about 20 minutes for the docking Benchmark 4.0, using 15° angular sampling on a single 2.8 GHz 64-bit Opteron processor with 8 GB available RAM. ZDOCK uses either a 6° or 15° angular spacing, which explores 54,000 or 3,600 Euler angle sets, respectively. In the current work, we adopted 68,760 and 4,392 angle sets for 6° and 15° angular spacing respectively, in order to achieve a more uniform coverage of the angular space [27], [28]. The coordinates of atom *k* of ligand prediction *i* are related to its Euler angles *ψ^i^*, *θ^i^*, and *φ^i^* and the starting ligand coordinates (labeled as prediction *0*) through the rotation matrix **T**:
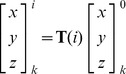
(2)

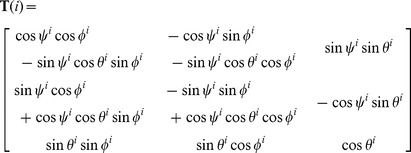
(3)


### Dataset

The complexes for testing and training were obtained from the widely used protein-protein docking benchmark developed by our lab (version 4.0) [29]. The benchmark contains 176 protein-protein complexes of which both the bound and unbound structures are available, and is non-redundant at the SCOP [30] family-family pair level. According to biochemical function, 52 complexes are of the enzyme-inhibitor type, 25 are antibody-antigen, and 99 ‘others’. In addition, the complexes are classified according to expected docking difficulty.

We consider a docking prediction a *hit* when the interface Cα atoms of the complex have a root-mean-square-distance of less than 2.5 Å from the native (bound) complex. Generally we assess the performance of a docking algorithm using the *success rate* (SR) and *average hit count* (AHC) curves. The success rate is the fraction of test cases that have at least one hit, as a function of the number of allowed predictions for each test case. The average hit count is the total number of hits as a function of the number of predictions considered for each test case, divided by the total number of test cases. Often it is desired to represent the performance of an algorithm by a single number, instead of a graph that needs visual inspection. Here we use the *integrated success rate* (ISR) [8], which is obtained from plotting the success rate against the log of the number of predictions for the range 1–1000, with the ISR defined as the area under the success rate curve normalized to 1. The worst performance is at ISR = 0, and perfect performance is at ISR = 1.

For the optimization of the weights in the section that combines funnel properties and ZDOCK score, we performed 22-fold cross-validation for training and testing. The target function in the optimization is the ISR.

### Clustering

The purpose of clustering or pruning a set of docking results is two-fold. First, removing predictions that are similar (or redundant) to others reduces the set of predictions that needs to be considered further. Second, the density of a prediction, defined as the number of predictions that are similar to the prediction, may indicate whether the prediction is correct.

We first prune using an iterative algorithm. The center of the first cluster is the complex with the highest ZDOCK score. We then eliminate all the predictions that are similar to this prediction, based on some similarity measure (RMSD or angular distance in this work), using a specified cutoff. Of the remaining set, the prediction with the highest score becomes the center of the second cluster, and these steps are repeated until no predictions remain in the list. The resulting set of cluster centers represents a pruned set of predictions, which are spaced by at least the threshold. The clustering process is finalized by determining how many predictions of the original set are within the threshold distance of each cluster center.

For the pruning using angular distance we also explored a ‘translation-restricted’ variant of the algorithm. Predictions that have a translational difference of more than half the receptor size are not allowed to be in the same cluster, as they are highly unlikely to belong to the same funnel. The translational difference is obtained from the three translational coordinates in the rigid-body docking, and the receptor size is defined as the average of the lengths of the protein in the directions of the three Cartesian axes. Because the translational difference is needed only for pairs of predictions that have angular distances under the angular threshold, this extension to the algorithm only increases the computational time moderately.

An alternative approach to score-based pruning is to rank and prune based on the density of predictions. We explored two versions of density-based pruning. First we followed the ClusPro algorithm [31], which determines for each prediction the number of neighbors within a threshold distance, ranks accordingly, and uses this rank for a pruning step. Second, we used R to hierarchically cluster the predictions, and varied the height at which the branches are cut to find the best performance. For both density-based algorithms we used the top scoring 2000 predictions as starting point, and tested both RMSD and angular distance. The ZDOCK score was used to rank predictions that have identical densities. For the hierarchical clustering we used the complete linkage method, and the defined the medoid as the prediction that represents a cluster.

### Funnel Analysis

We analyze the energy funnel around each prediction using angular distances and RMSD’s. For each prediction, we plot the docking scores of the N most similar predictions as a function of either angular distance or RMSD from the prediction. Using linear regression, we then determine the slope and intersect of the best-fit line of the plot and use them to characterize the energy funnel around the prediction in question. In addition, we calculate the average docking score of the N most similar predictions.

### Angular Distance

In this work we use the angular distance as a measure of the similarity of two docking predictions. In our docking algorithm, the rotation of the ligand from its original coordinates is described by three successive rotations, represented by the Euler angles. The total angle resulting from the three successive rotations, however, is not simply the sum of the three Euler angles, nor is it the Pythagorean distance (as the three rotations are not orthogonal). The Euler representation is equivalent to the axis-angle representation, which rotates the object about a single vector in the 3D space. Because the direction of this vector can be described using two variables, the axis-angle representation has three independent variables (the same number as the Euler representation). The angular distance α of a 3D rotation is equivalent to the angle in the axis-angle representation and is related to the trace (tr) of the rotation matrix **T**:
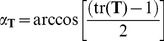
(4)


Thus we can express the rotation of a docking prediction specified by three Euler angles as a rotation matrix, from which we can then obtain the angular distance α between this prediction and the starting ligand orientation.

The angular distance between the rotations of two docking predictions *i* and *j*, which are specified by two rotation matrices **T** *^i^* and **T** *^j^* respectively, is defined as:
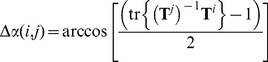
(5)


The inverted matrix **T** ^−1^ (which for rotation matrices is identical to the transpose) is the rotation in the opposite direction of **T**. The product (**T** *^j^*)^−1^ **T** *^i^* specifies the rotation needed to generate prediction *j* starting from prediction *i*. Equation 5 is an analytical expression and can be evaluated rapidly. Because we use fixed sets of angles in our docking algorithm ZDOCK (thus with fixed rotation matrices **T**), we can pre-compute the lists of the closest neighbors for each rotation and use the results to evaluate the predictions of any docking run.

## Results

**Figure 1:**
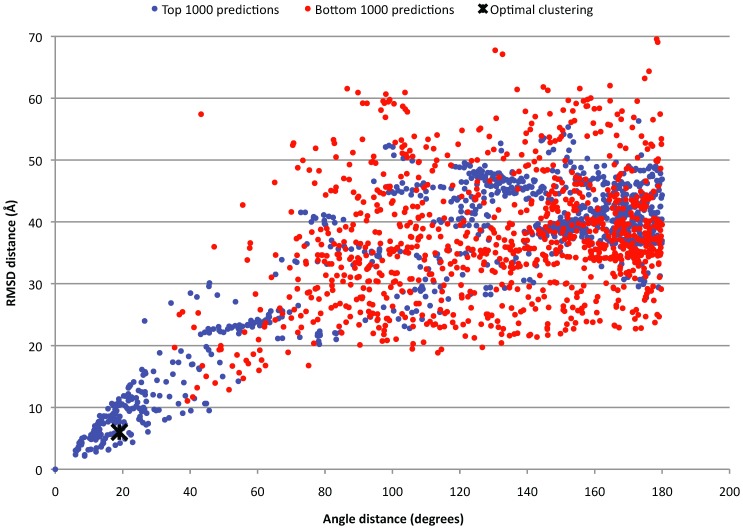
Angular and RMSD distances between the first ZDOCK prediction of 1BJ1 and the top 1000 and bottom 1000 predictions. The optimal clustering corresponds to 19° angular distance pruning and 6 Å RMSD pruning.

In Figure 1 we plot the RMSD against the angular distance between the top ZDOCK prediction of the 1BJ1 complex and 2000 predictions (top 1000 and bottom 1000 according to ZDOCK score). We use this complex as an example because its top ZDOCK prediction is the closest to the native complex of all test cases in our benchmark. It is clear that the angular distance and RMSD are correlated. The correlation is particularly strong for shorter distances, which is the region that we are concerned with for most purposes.

### Hybrid-resolution Docking

**Figure 2:**
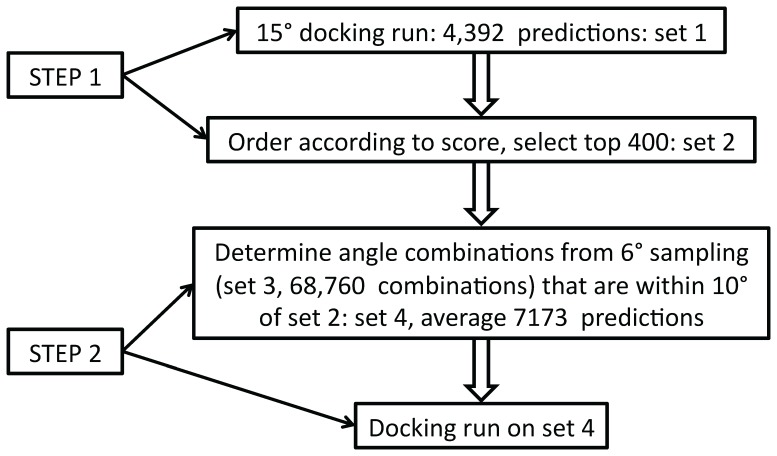
Schematic overview of the hybrid-resolution approach.

We explored the possibility of reducing the computational cost of protein-protein docking by an approach consisting of two stages with different angular resolutions (Figure 2). A two-stage approach with different *translational* resolutions was explored previously in context of rigid-body protein-protein docking by Vakser and coworkers [4]. We argue that a first low-resolution stage can identify the regions in the angular space that contain near-native predictions. The second stage then refines the most promising regions using high-resolution sampling. Here we show results of a hybrid 15°/6° run. For each complex, we first took the 400 top predictions from a 15° sampling run. This corresponded to roughly 10% of the total number of 4392 predictions. We followed this with a 6° sampling run in which we only considered those angle sets that were within 10° of the 400 predictions identified in the first stage. Generating this reduced 6° angle list is computationally inexpensive as we used pre-computed lists of nearest neighbors based on angular distance defined by Equation 5. The average number of angle sets retained in the 6° run was 7173, resulting in an average total number of 11,565 angle sets (4392+7173) that needed to be evaluated. This corresponds to 17% of the angle sets of a standard 6° sampling run (68,760 angle sets), or a 6-fold reduction in total computational time.

**Figure 3:**
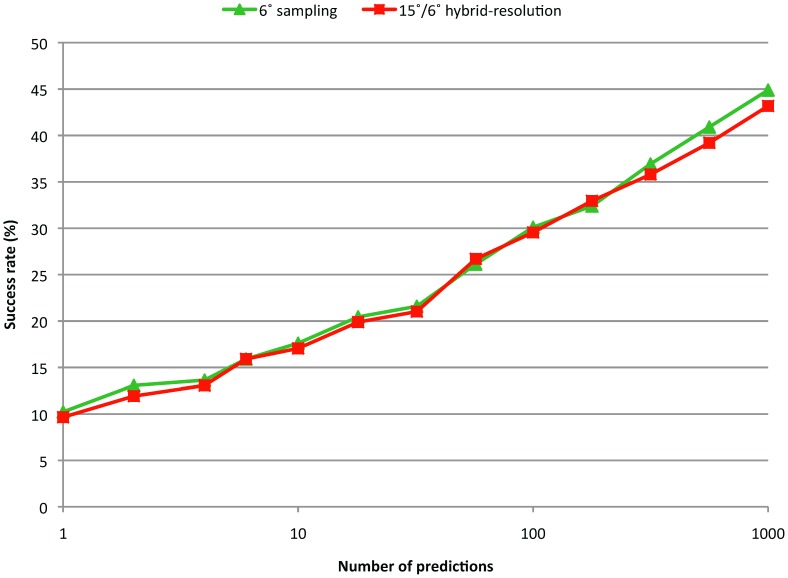
Success rate for the standard 6° rotational sampling and the hybrid-resolution approach.

**Figure 4:**
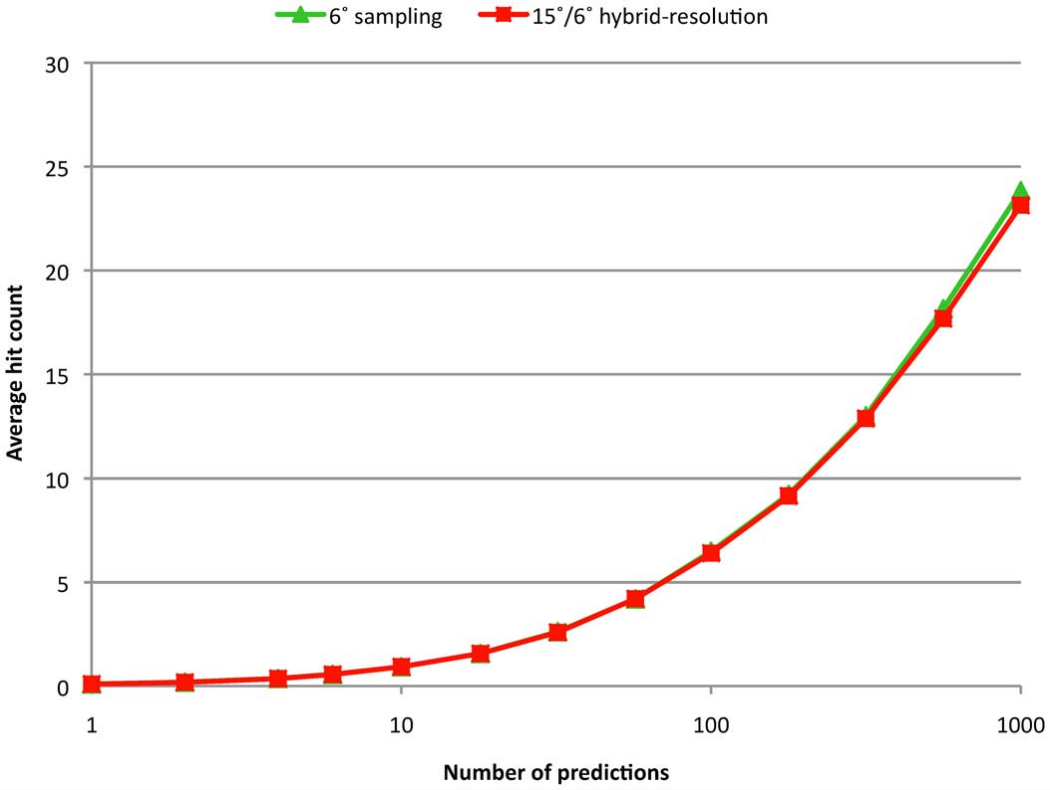
Average hit count for the standard 6° rotational sampling and the hybrid-resolution approach.

Figure 3 shows the SR of both the standard 6° sampling and the 15°/6° hybrid-resolution runs. The performances are nearly identical, with ISR = 0.239 for the hybrid-resolution and 0.241 for the standard 6° sampling run. Figure 4 shows the AHC, which is also nearly identical for the standard and hybrid-resolution runs. Previously we showed that there was a tradeoff between SR and AHC: decreasing the total number of predictions increases the SR and decreases the AHC and vice versa [20]. However, we see from Figures 3 and 4 that with the hybrid-resolution approach we can reduce the number of predictions by a factor of about 10 compared with a standard 6° sampling run while maintaining the same performance as measured by SR and AHC.

**Figure 5:**
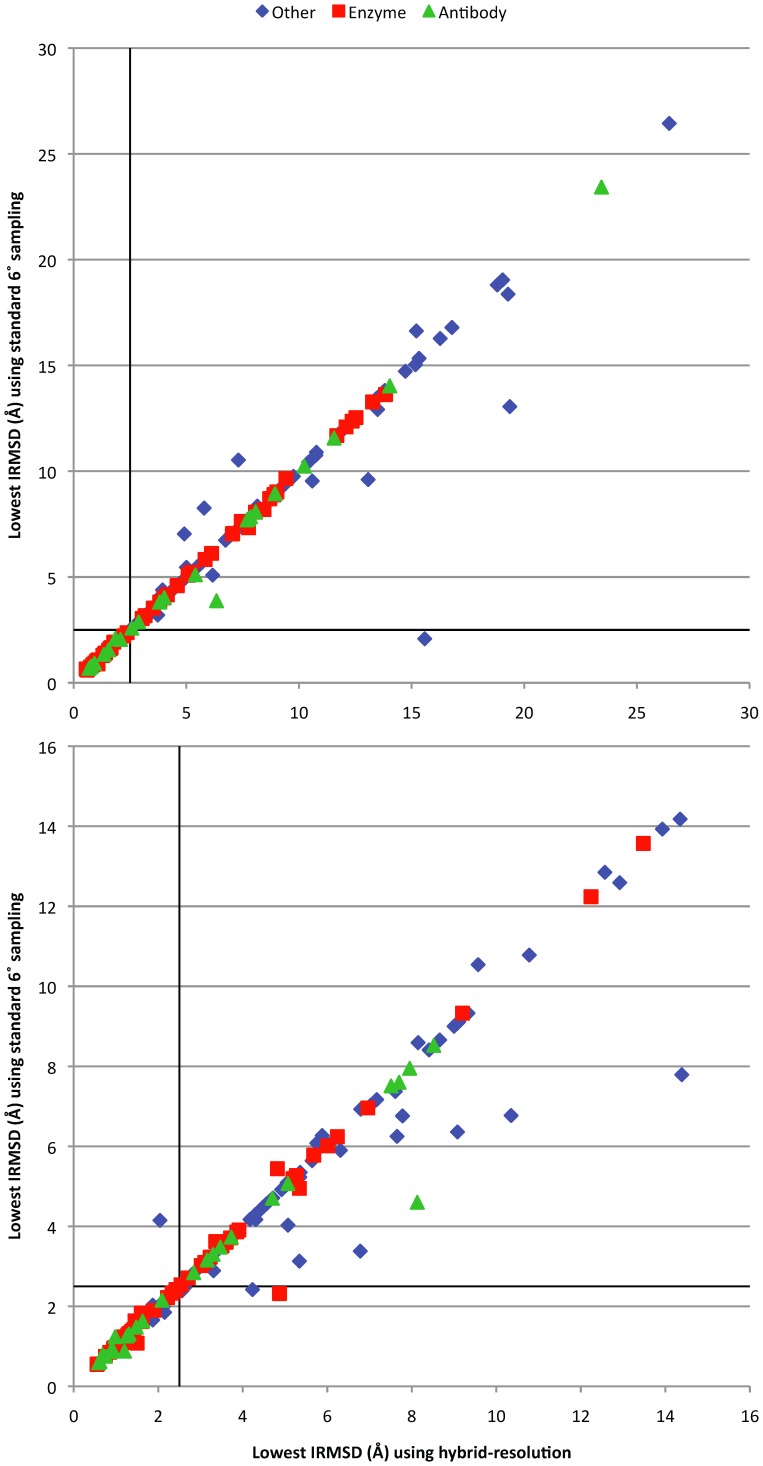
IRMSD’s of the best predictions for the standard 6° rotational sampling run and the hybrid-resolution run, for the top 100 (top panel) and top 1000 (bottom panel) predictions.

To further analyze the performance of the hybrid-resolution approach, we compared for each complex in our test set the best prediction obtained using the standard approach (uniform 6° rotational sampling) with the best prediction obtained using the hybrid-resolution approach. The best prediction of a set is defined as that with the lowest interface RMSD (IRMSD) from the bound complex [5]. In Figure 5 we show the best prediction among the top 100 and the top 1000 predictions (by ZDOCK score) respectively, for each test case. We see that for both the top 100 and the top 1000 predictions, most of the IRMSD’s lie on the diagonal, which indicates that the best predictions of the two approaches are very similar. For the top 100 predictions (Figure 5 top), the best predictions obtained with the two approaches differ only for a few test cases, mostly from the ‘others’ category. The overall performance is very similar, indicated by the similar number of points above and below the diagonal. Only one test case has a hit for the standard approach and not for the hybrid-resolution approach. Thus for this case the near-native region of the 6D space is not sampled in the top 400 predictions of the first stage, which may be due to the score being sensitive to small perturbations. For the top 1000 predictions, we see that when the IRMSD’s are different, they are generally lower using the standard approach. However, we see that only three hits (out of 176 test cases) are not shared by the two approaches, with two hits generated by the standard approach only and one hit generated by the hybrid-resolution approach only. These results show that the two approaches generally make highly similar predictions.

### Pruning and Clustering

**Figure 6:**
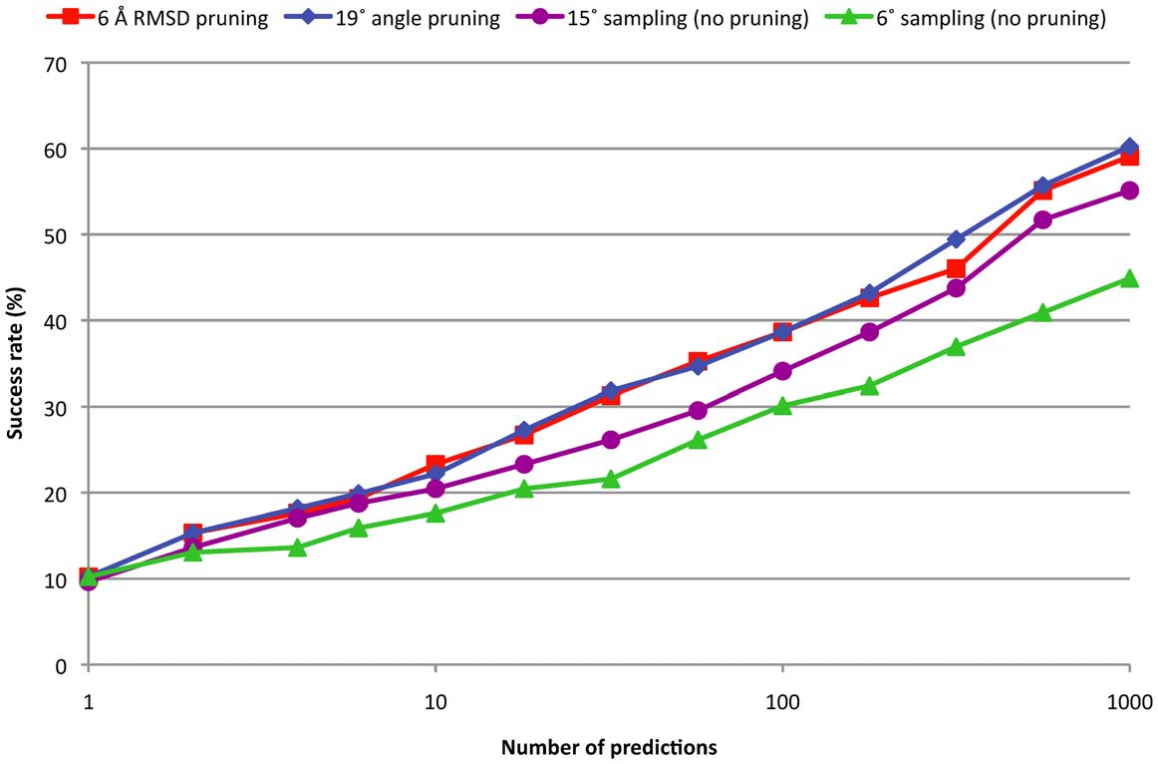
Success rate for 15° and 6° rotational sampling, and for 6° rotational sampling with 19° angular distance pruning or 6 Å RMSD pruning.

**Figure 7:**
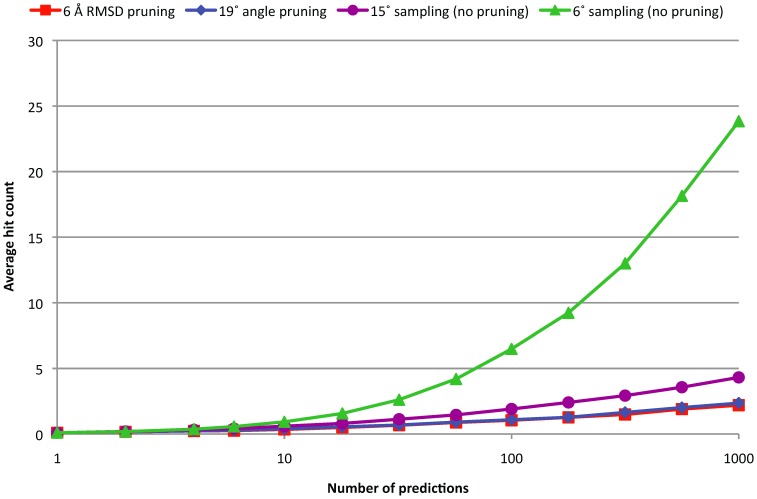
Average hit count for 15° and 6° rotational sampling, and for 6° rotational sampling with 19° angular distance pruning or 6 Å RMSD pruning.

Protein-protein docking algorithms typically make a large number of predictions, many of which are very similar. Therefore, before further refining the predictions, the set is usually *pruned* or *clustered* to remove the redundant predictions. Using our benchmark, we determined the threshold that maximizes the ISR for pruning using similarity based on RMSD and using similarity based on angular distance. For RMSD based pruning the optimal threshold was 6 Å and for angular distance pruning the optimal threshold was 19°. For these cutoffs, the angular distance and RMSD based pruning retain an average number of 1347 and 6316 predictions, respectively. We plot the point that corresponds to these optimal thresholds as an asterisk in Figure 1, and indeed find this point in the cloud that shows a strong correlation between angular distance and RMSD. The ISRs obtained using the optimal angular distance and RMSD based clustering are 0.320 and 0.313 respectively, both improved over the uniform 6° and 15° sampling (ISR = 0.241 and 0.287). Figures 6 and 7 show the success rates and average hit counts and the results are very similar between angular distance and RMSD, with angular distance slightly outperforming RMSD in SR and both reducing the AHC to almost the same level. This shows that for clustering, the angular distance is a suitable alternative for the generally used RMSD. To ensure that our docking algorithm is not biased toward our test cases, we repeated the analysis just for the cases that were newly introduced in the latest version of our Benchmark, which was published three years after the version of ZDOCK we used in this work. For the pruning with RMSD and with angular distance, we find ISRs of 0.280 and 0.270, respectively. Thus the performance with the two distance metrics is still very similar.

With the translation-restricted version of the angular pruning algorithm we obtain the best ISR with a threshold of 19°, which is the same as for the unrestricted algorithm. The ISRs of the unrestricted and restricted algorithms are very similar (0.320 and 0.318, respectively), which indicates that the funnels for the top predictions are generally well defined and the angular distance is a good approximation for the distance in 6D space.

For the density-based clustering, the number of predictions retained after pruning may be small because we start with a set of only 2000 predictions. Therefore we used the top 10 to assess the performance. Furthermore, we found that the ISR is very sensitive to small differences in rank when only the top 10 is considered. Consequently, we used the top 10 *success rate* (ranging from zero to one) to assess the performance of the density-based clustering. Using the ClusPro approach, we found best SRs of 0.222 and 0.227 using RMSD and angular distance, respectively, at thresholds in the range of 5–7 Å and at 20°, respectively. Using hierarchical clustering, we found best SRs of 0.222 and 0.233 using RMSD and angular distance, respectively, with branch cutoff at heights in the ranges of 14–18 Å and 57–69°, respectively. Thus the RMSD and angular distance again yield similar performance, with the angular distance slightly outperforming RMSD. When we use the score-based pruning algorithm, we find top ten SRs of 0.233 using both RMSD and angular distance.

### Funnel Analysis

**Figure 8:**
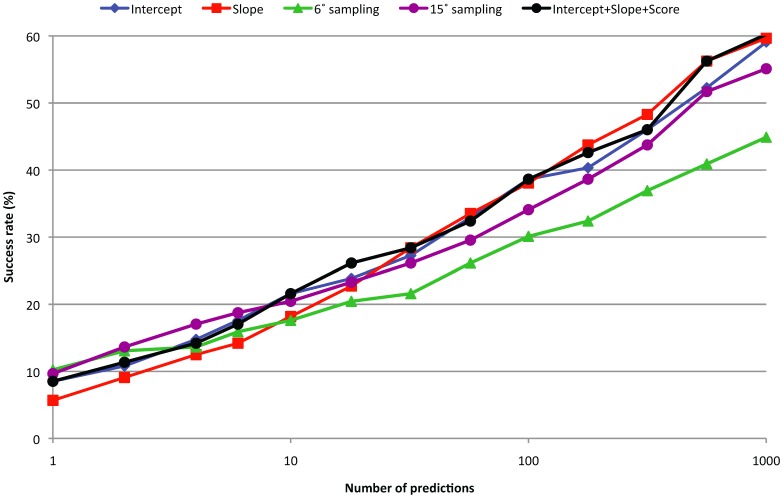
Success rate for 15° and 6° rotational sampling, the Intercept and Slope funnel properties (based on 10 closed neighbors using angular distance), and the scores and properties combined in a weighted linear function (training and testing using 22-fold cross validation).

**Figure 9:**
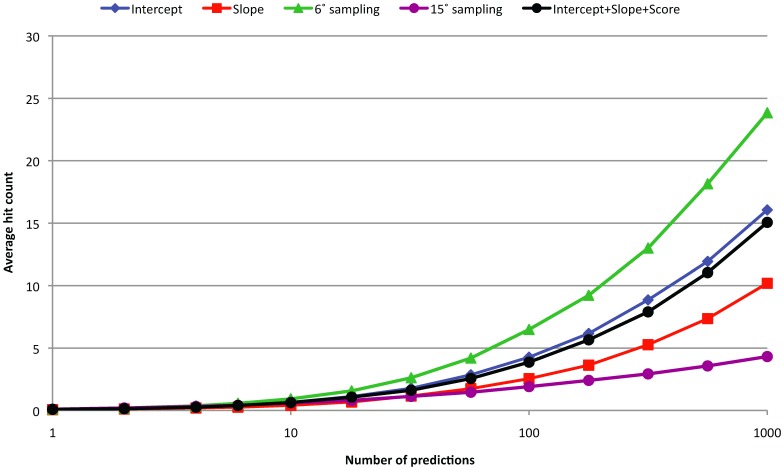
Average hit count for 15° and 6° rotational sampling, the Intercept and Slope funnel properties (based on 10 closed neighbors using angular distance), and the scores and properties combined in a weighted linear function (training and testing using 22-fold cross validation).

A collection of predictions that are similar (typically defined as low RMSD’s) can contain more information than a single prediction. In its simplest form, one can average the scores of grouped predictions to alleviate random errors. More complex is to analyze the relationship between the predictions, specifically whether they form a ‘funnel’ where the best prediction is assumed to be at the center. Here we analyze funnels in a simple way by calculating the slope and intersect (using linear regression) of a collection of nearest neighbors of a given prediction. A larger slope means a better-defined funnel, and the intersect is an estimate of the score at the center of the funnel. In Table 1 we show the ISR’s obtained with angular distance and RMSD while varying the total number of predictions used to characterize the funnel. RMSD and angular distance show similar behaviors, but angular distance shows the best performance. The ZDOCK score on its own with 6° and 15° sampling gives ISR = 0.241 and ISR = 0.287, respectively. Thus by taking into account either RMSD or angular distance funnel, we can obtain an improvement over the raw scores. In the Figures 8 and 9 we show the performance of the funnels using angular distance, 6° sampling, and 10 neighbors. We also constructed a weighted linear combination of the ZDOCK score and the intercept and slope funnel properties, which gives ISR = 0.300.

**Table 1:** ISR’s for funnel properties obtained using angular distance or RMSD.

	Angular	Angular	Angular	RMSD	RMSD	RMSD
N	Intercept	Slope	Average score	Intercept	Slope	Average score
5	0.072	0.062	0.210	0.200	0.202	**0.216**
10	**0.293**	**0.290**	**0.212**	0.239	0.245	0.209
15	0.255	0.248	0.209	**0.244**	**0.252**	0.199
20	0.247	0.251	0.206	0.237	0.242	0.198
30	0.236	0.236	0.202	0.237	0.249	0.192
50	0.228	0.233	0.196	0.228	0.236	0.184
100	0.218	0.223	0.188	0.212	0.229	0.173
150	0.215	0.219	0.181	0.204	0.223	0.166
200	0.213	0.217	0.176	0.200	0.214	0.164

N is the number of the closest neighbors used to calculate the properties. The best prediction for each property is in bold.

## Discussion and Conclusions

In this work we explored the use of angular distance in protein-protein docking to measure similarities of predictions. Compared with RMSD, angular distance represents a reduction from six dimensions to three dimensions. Because the angular distances in a docking run are known *a priori* they can be used in a hybrid-resolution scheme. We showed such a scheme that on average reduces the computational cost of a docking run by a factor of six while maintaining the success rate and average hit count compared with a standard 6° docking run. Our results suggest that the energy landscape for protein-protein binding computed using angular distance is reasonably smooth, because the best orientations that can be identified by a denser sampling (6° in our case) are in the vicinity of the best orientations identified by a coarser sampling (15° in our case).

We also found that angular distance performed slightly better than the RMSD for funnel analysis, despite the fact that RMSD is a more accurate measure than angular distance for the distance between predictions. Specifically, when we define a funnel using the N closest neighbors based on angular distance, some of these neighbors can have large RMSD’s to the prediction in the center of the funnel and may not even belong to the same funnel were the non-reduced 6D space considered. However several reasons prevent such situations from affecting the performance of funnel analysis. In the example in Figure 1 we see that the angular distance and RMSD are well correlated for predictions within the funnel. We reason that the deeper an energy funnel, the stronger the correlation between angular distance and RMSD. Because the deepest funnels dominate the docking performance, the inaccuracy of angular distance has little impact on the performance. Furthermore, in some cases, RMSD may be overly sensitive to small structural differences and the angular distance avoids this by lowering the dimensionality (three vs. six degrees of freedom), hence its better performance.

Furthermore, we found that a simple pruning algorithm with angular distance performed slightly better in terms of ISR than the same algorithm with RMSD. Moreover, for the best angular distance-based pruning far fewer predictions were retained (19° cutoff, average 1347 predictions retained) than with the best RMSD-based pruning (6 Å cutoff, average 6316 predictions retained). This is probably because some predictions that are similar based on angular distance can be very different in the 6D space and these predictions are pruned out by angular distance but not by RMSD. Because the two approaches retain the same number of hits (Figure 7), angular pruning enriches hits by nearly five fold and can benefit downstream analysis.

## Acknowledgments

We thank Julie Mitchell (University of Wisconsin – Madison) for providing the angle sets used in this work, and Brian Pierce (University of Massachusetts Medical School) for helpful discussions.
